# A Modern Approach to Clinical Outcome Assessment in Allergy Management: Advantages of Allergen Exposure Chambers

**DOI:** 10.3390/jcm13237268

**Published:** 2024-11-29

**Authors:** Magdalena Zemelka-Wiacek

**Affiliations:** Department of Clinical Immunology, Faculty of Medicine, Wroclaw Medical University, 50-367 Wrocław, Poland; magdalena.zemelka-wiacek@umw.edu.pl; Tel.: +48-506-224-077

**Keywords:** allergen challenge, allergen exposure chamber, allergen immunotherapy, allergic rhinitis, allergic rhinoconjunctivitis, environmental chamber

## Abstract

Allergic diseases triggered by airborne allergens such as allergic rhinitis and conjunctivitis are increasingly prevalent, posing significant challenges for both patients and healthcare systems. Assessing the efficacy of allergen immunotherapy and other anti-allergic treatments requires precise and reproducible methods. Allergen exposure chambers (AECs) have emerged as advanced tools for evaluating clinical outcomes, offering controlled conditions that address many limitations of traditional field-based studies. This review explores the advantages of AECs in allergy management, emphasizing their role in providing standardized allergen exposure for both clinical research and routine assessments. AECs deliver consistent and reproducible data comparable to the nasal allergen challenge and natural allergen exposure, making them a valuable addition to the diagnosis and treatment effectiveness of allergic diseases. Although they are well suited to early-stage clinical trials, further standardization and validation are needed to gain broader acceptance in pivotal phase III studies. Future research should focus on refining AEC protocols and integrating them into regulatory frameworks, ensuring their role in the advancement of therapeutic approaches for allergic diseases.

## 1. Introduction to Allergen Exposure Chambers (AECs)

In the routine care of patients with allergic rhinitis or rhinoconjunctivitis and during clinical trials for allergen immunotherapy (AIT), precise outcome assessments are critical to evaluating the effectiveness of treatments, understanding disease progression, and making informed clinical decisions [1,2,3]. These assessments provide the quantitative and qualitative data needed to ensure that therapeutic interventions achieve their intended outcomes and meet regulatory standards [4]. Inaccurate or imprecise measurements can lead to the misinterpretation of results, affecting patient safety, treatment efficacy, and overall healthcare quality. Traditionally, outcome assessments have relied on a combination of clinical observations, patient-reported outcomes, and laboratory tests [5,6].

Allergen exposure chambers (AECs), also referred to as environmental (exposure) chambers/units or allergen challenge facilities/chambers, represent a significant advancement in the precision and control of allergen challenges used in clinical and laboratory outcome assessments, particularly in allergy and respiratory research [7]. Similar to the nasal allergen challenge (NAC), tests conducted in AECs are allergen-provocation tests, designed to provoke specific physiological responses in a safe, reproducible manner. AECs are highly controlled facilities where specific allergens can be introduced at known concentrations to study their effects on participants. This controlled exposure allows for consistent and reproducible assessments, eliminating many of the variables that complicate traditional outcome measurements [8]. Moreover, the One Health approach (the interconnectedness of human, animal, and environmental health) recognizes the importance of environmental factors, such as air quality and allergen exposure, in the development and management of allergic diseases, further highlighting the utility of AECs in exploring these connections [9].

**Figure 1:**
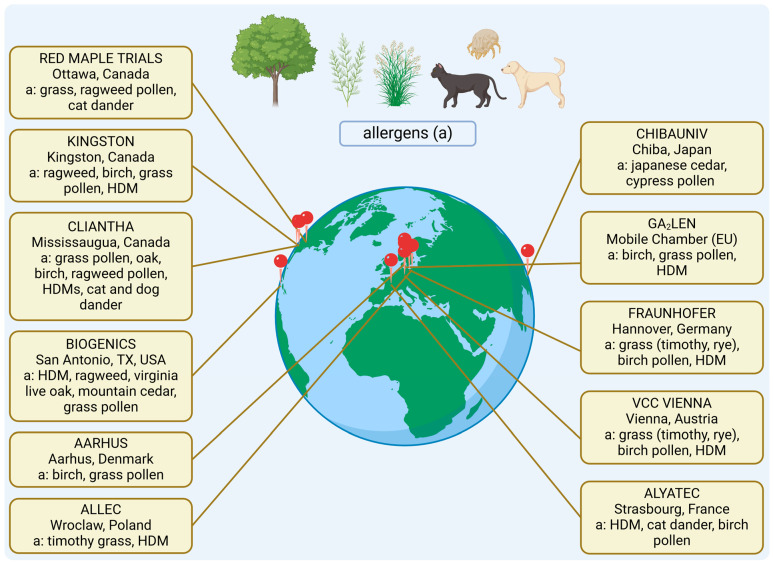
Allergen exposure chambers (AECs) around the world [14]. This map displays the global distribution of AECs and the specific allergens (a) used in their controlled exposure studies. The diversity of allergens allows for targeted research and treatment trials tailored to regional allergic sensitivities. Note: VVC VIENNA was the first chamber in Europe with 38 years of experience, now closed.

The evolution of AECs began with the need to create standardized environments for testing the efficacy of ATI and other treatments for allergic diseases. Early models of AECs were relatively simple and focused primarily on simulating natural allergen exposure conditions [10,11]. Over time, advancements in technology and a deeper understanding of allergen dynamics have led to the development of sophisticated AECs that can precisely control temperature, humidity, pressure, and allergen concentration [12,13]. These chambers are now capable of simulating various environmental conditions, making them invaluable tools not only for allergy research but also for broader applications in respiratory and immune response studies (Figure 1).

In the context of clinical and laboratory assessments, AECs offer several advantages. They provide a platform for the real-time monitoring of physiological responses to allergens under controlled conditions, which can be particularly useful for studying the mechanisms of action of new therapeutic agents [15]. Furthermore, AECs enable researchers to conduct longitudinal studies with consistent allergen exposure, which is essential for evaluating treatments’ long-term efficacy and safety. This level of control and precision is difficult to achieve with traditional methods, making AECs an important component of modern clinical and laboratory outcome assessment [16,17].

## 2. Types of EACs

### 2.1. Clinical Endpoints During the Allergen Challenge

**Figure 2:**
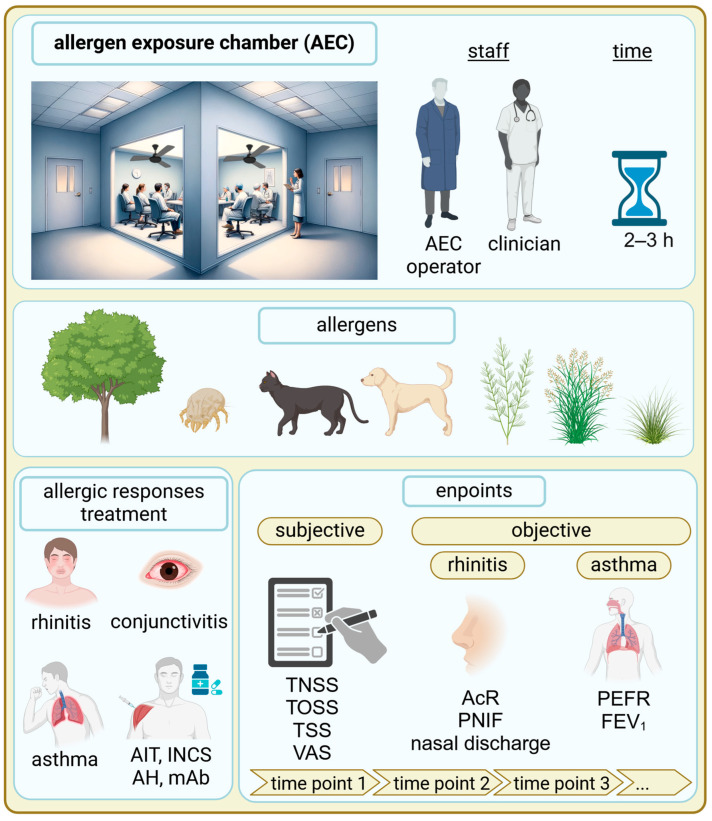
Overview of allergen AEC setup and clinical assessment. It highlights the role of AEC staff and the typical duration of allergen challenges. Various allergens used to provoke allergic responses. The process involves measurements at multiple time points to monitor changes throughout exposure. AECs can be utilized for the diagnosis of conditions like allergic rhinitis, conjunctivitis, and asthma and for assessing anti-allergic therapies (intranasal corticosteroids, antihistamines, and biologic therapy with monoclonal antibodies) or AIT efficacy. Abbreviations: AEC = allergen exposure chamber; AcR = acoustic rhinometry; AH = antihistamines; AIT = allergen immunotherapy; FEV1 = forced expiratory volume in 1 s; INCS = intranasal corticosteroids; mAb = monoclonal antibody; PEFR = peak expiratory flow rate; PNIF = peak nasal inspiratory flow; TNSS = total nasal symptom score; TOSS = total ocular symptom score; TSS = total symptom score; VAS = visual analog scale.

AECs provide a precise measurement of clinical endpoints in response to defined allergen exposures. Typically, these challenges last between 2 and 4 h (however, some might even take up to 6 h), with parameters measured before, during, and after exposure to assess the onset and duration of symptoms. At one time, there are typically 10 to 50 patients in the chamber, depending on the specific AEC used [18,19,20,21,22,23,24]. These endpoints allow for a comprehensive evaluation of allergic reactions in a consistent manner that is often challenging to achieve in field studies due to variability in environmental allergen exposure [25] (Figure 2)

A range of both subjective and objective endpoints can be assessed during controlled challenges. Subjective measures include the total nasal symptom score (TNSS) and the visual analog scale (VAS), which quantify subjective symptoms such as nasal congestion, sneezing, itching, and discharge, as reported by patients, or other symptom scores [26,27,28,29,30]. TNSS provides a cumulative score of nasal symptoms, while VAS allows patients to rate symptom severity on a scale. Objective endpoints include measurements like the following: (a) acoustic rhinometry, which evaluates nasal airway patency by measuring the cross-sectional area of the nasal passage, providing insights into nasal obstruction; (b) peak nasal inspiratory flow (PNIF), which measures the maximum speed of inhalation through the nose, reflecting airflow limitation; (c) and nasal secretion weight, which quantifies the volume of nasal discharge, offering a direct measure of symptom severity [31,32,33,34,35,36].

Lung function parameters include the peak expiratory flow rate (PEFR) and forced expiratory volume in the first second (FEV1). PEFR assesses the highest flow achieved during forced expiration, while FEV1 measures the volume exhaled during the first second of a forced breath. These parameters are widely recognized as objective measures in asthma assessments (see Section 4). Additionally, they can serve as safety indicators in studies primarily involving patients with rhinitis or rhinoconjunctivitis without a confirmed diagnosis of asthma [37,38,39]. These endpoints allow for a comprehensive evaluation of allergic reactions in a consistent manner that is often challenging to achieve in field studies due to variability in environmental allergen exposure.

Adverse events in AECs are generally rare and mild due to the controlled nature of the environment [33,40]. Commonly reported events include mild nasal irritation, sneezing, or watery eyes, typical allergic responses to the allergens being tested, as well as asthma episodes or headaches [34,41,42]. Serious adverse reactions, such as anaphylaxis, are exceedingly uncommon due to careful participant screening and the use of standardized allergen doses [43].

### 2.2. Standardization and Validation

The validation of AECs is critical to their use in clinical trials and allergy research. Validation ensures that the allergen exposures in AECs are consistent and that the measured responses are reproducible across different studies. AEC validation involves confirming that environmental conditions such as temperature, humidity, and allergen concentration remain stable throughout the exposure period, which is crucial for reproducible outcomes [44,45,46,47,48,49,50,51,52,53]. For instance, studies have shown that stable allergen concentrations, such as house dust mite (HDM) levels in the range of 1000 to 8000 particles per cubic meter, can induce consistent symptoms in allergic rhinitis patients, with minimal variation across multiple trials [31]. These validations align with guidelines from the European Academy of Allergy and Clinical Immunology (EAACI), which emphasize the importance of standardizing AEC procedures to ensure comparability between studies conducted in different facilities [8]. Overall, validated AECs represent a reliable tool for evaluating new treatments and understanding allergic responses in a controlled and reproducible manner.

### 2.3. Diverse Allergen Models

Various AEC models have been developed to expose patients to allergens such as pollen, dust mites, and animal dander, some of them listed below; but AEC can also reliably simulate weather conditions to induce symptoms in nonallergic rhinitis (NAR) patients, providing a valuable tool for diagnosing NAR and exploring new therapeutic approaches [54].

#### 2.3.1. Birch Pollen

Research on birch pollen allergy has explored various aspects of allergic reactions and the efficacy of treatments [55,56,57]. One study investigated the effects of a daily symbiotic food supplement in patients with birch pollen-induced rhinoconjunctivitis, finding significant improvements in symptom scores and well-being after 4 months of supplementation, with no adverse events and enhanced tolerance to birch pollen exposure [37]. Another study evaluated the non-inferiority of N-acetyl aspartyl glutamic acid compared to fluorometholone for treating birch pollen-induced allergic conjunctivitis, finding that both treatments similarly delayed the onset of conjunctival responses [58]. A different study examined the symptom response to birch pollen exposure in subjects who were allergic to oak but had no previous natural exposure to birch, finding that these birch-naive subjects displayed symptom severity similar to those with prior birch exposure, highlighting the cross-reactivity between oak and birch pollen allergens in an allergen challenge chamber [59].

#### 2.3.2. Cat Dander

A naturalistic exposure chamber was used to simulate a real-world environment where live cats reside, allowing for a more authentic allergen exposure experience for cat-allergic subjects during controlled trials [41]. A recent investigation evaluated the efficacy of air cleaners in preventing early and late asthmatic responses in cat-allergic patients, finding that active air cleaners significantly reduced the incidence and severity of these responses compared to placebo and protected against indoor airborne allergens [60,61]. The effect of omalizumab, an anti-IgE monoclonal antibody (mAb), in preventing acute bronchoconstriction during controlled cat-allergen exposure was demonstrated by its significant reduction in the decline in lung function (FEV1) and improved tolerance for cat allergens compared to placebo. This highlights its efficacy in mitigating asthma symptoms during allergen challenges [62].

#### 2.3.3. House Dust Mite (HDM)

The effect of a barrier-forming, drug-free nasal spray was evaluated, demonstrating its ability to significantly reduce allergic rhinitis symptoms triggered via HDM allergens, with protection lasting up to 180 min and showing a good safety profile [63]. The effect of using hypertonic sodium chloride as a carrier for HDM allergens was evaluated, demonstrating its noninferiority compared to lactose in delivering HDM, making it a cost-effective and practical alternative for allergen delivery in controlled chamber studies [27]. There are relatively few studies that examine laboratory parameters in controlled allergen exposure settings. The study on biologic responses assessed changes in blood markers following allergen exposure. Fifty-five participants underwent the challenges, with blood samples taken before and after exposure. The results showed significant increases in white blood cell, neutrophil, and lymphocyte counts post-exposure, with allergic participants also exhibiting changes in specific immunoglobulin E (IgE) levels and reduced eosinophil counts. Notably, serum interleukin (IL)-13 decreased in allergic individuals, while tumor necrosis factor-alpha (TNF-α) levels dropped in non-allergic participants, highlighting the immune response dynamics during the challenge [64].

#### 2.3.4. Japanese Cedar Pollen

The efficacy and safety of bepotastine (antihistamine) in suppressing nasal symptoms induced via Japanese cedar pollen were evaluated in a randomized, placebo-controlled study, showing significant reductions in symptoms during exposure in AEC [65]. The exposure to Japanese cedar pollen revealed that nasal symptoms peaked after 2 h and persisted for over 6 h, with increased release of inflammatory mediators, including the concentrations of histamine, tryptase, IL-5, -3, -33, and -31, and substance P increased over time, whereas that of nasal fractional exhaled nitric oxide (FeNO) decreased [66]. A study investigated the impact of testing periods on pollinosis symptoms using an AEC, revealing that symptoms were more severe following repeated exposures and immediately after the pollen season. This underscores the importance of timing for the accurate assessment of symptoms [67].

#### 2.3.5. Ragweed Pollen

The evaluation of drugs for seasonal allergic rhinitis and related conditions through controlled AEC allows for precise measurement of efficacy and onset of action. For instance, studies such as the one assessing the onset of action of loratadine (antihistamine) tablets demonstrated significant symptom relief post-dose using a controlled ragweed pollen exposure model [68,69,70,71]. Another study evaluated the efficacy of fexofenadine–pseudoephedrine, an antihistamine–decongestant combination that offers broader symptom relief for individuals experiencing both allergy symptoms and nasal congestion, in a controlled setting. The study demonstrated a rapid onset of action, with a significant reduction in allergy symptoms observed within 45 min [72,73]. Additionally, controlled exposure studies have been used to validate AIT for ragweed-induced allergic rhinitis; patients receiving immunotherapy showed significantly reduced symptoms compared to those without [74]. Also, different drugs were tested, including mometasone furoate (glucocorticosteroid spray), fexofenadine hydrochloride (second-generation antihistamine), reproxalap (small-molecule reactive aldehyde species inhibitor for the treatment of inflammatory eye conditions, such as dry eye disease and allergic conjunctivitis) [75,76,77]. Such research underscores the importance of allergen chambers in optimizing therapeutic approaches and understanding drug performance under controlled conditions.

#### 2.3.6. Grass Pollen

The effect of wearing face masks, including medical and FFP2 masks, on preventing pollen-induced symptoms was evaluated in an exposure chamber, showing that both types of masks significantly reduced nasal and eye symptoms in individuals with grass pollen allergies, making them a viable non-medication option for symptom relief [78]. The study evaluated the specificity and reproducibility of nasal biomarkers in allergic rhinitis patients following controlled grass pollen exposure, finding consistent increases in eosinophils, IL-5, IL-6, and macrophage inflammatory protein 1β levels [79]. The dose-response relationship of a new timothy grass pollen allergoid was compared to a six-grass pollen allergoid, and it was found that both performed significantly better than placebo in reducing late-phase reactions, with the standard dose of the new preparation showing comparable efficacy to the six-grass preparation [80]. The timothy grass challenge has been successfully used to test the efficacy of various anti-allergic drugs, including cetirizine (an antihistamine) combined with pseudoephedrine (a decongestant), orastemizole (an older antihistamine), or intranasal glucocorticosteroids; additionally, a drug-free nasal spray containing bentonite was shown to reduce symptoms in patients with grass pollen exposure [42,81,82,83].

## 3. The Role of AECs in Assessing the Efficacy and Enhancing Clinical Trials for Allergen Immunotherapy and Other Anti-Allergic Treatment

AECs have become a useful tool in AIT research, providing a highly controlled and reliable environment for evaluating the efficacy of treatments. Their primary advantage lies in the ability to replicate environmental allergen exposure under standardized conditions, which enables researchers to consistently assess immune responses, treatment outcomes, and the onset of action for new therapeutic agents [84]. One of the key advantages of AECs is their capacity to reduce external variables that could affect the outcome of field-based trials. For example, in traditional field studies, participants may experience varying degrees of allergen exposure due to differences in geographical location, weather patterns, and pollen seasons, all of which can influence the severity of symptoms [85]. Confirmed insignificant differences in symptom severity between the two AECs, highlighting the feasibility of using multiple facilities for multicenter clinical trials [86]. By contrast, AECs ensure that each participant is exposed to the same controlled allergen concentration, providing a uniform baseline from which to assess the efficacy of AIT [87].

Numerous studies have evaluated the efficacy of allergen immunotherapy (AIT) for various allergens using AECs. For example, sublingual immunotherapy (SLIT) tablets for house dust mite (HDM)-induced allergic rhinitis have demonstrated dose-dependent symptom improvements, further supporting AIT as an effective therapeutic approach for HDM allergies [43,88]. Additionally, the SQ tree SLIT tablet demonstrated effectiveness in reducing rhinoconjunctivitis symptoms triggered by birch and oak pollen, with evidence of cross-reactive IgG4 induction across related species within the birch-homologous group [56]. Studies have also explored the use of timothy grass pollen SLIT for treating birch pollen-induced allergic rhinoconjunctivitis, though results highlighted the allergen specificity of this treatment [40]. Another study using AECs demonstrated the effectiveness of ragweed AIT in reducing ragweed-induced nasal symptoms, with participants in maintenance therapy experiencing significantly fewer symptoms compared to those without immunotherapy, though the effect on ocular symptoms was limited [74].

The efficacy of various anti-allergic treatments, including antihistamines, decongestants, and monoclonal antibodies, has been thoroughly evaluated using AECs. For instance, omalizumab has demonstrated its effect on mitigating asthma symptoms during controlled allergen challenges, significantly reducing lung function decline and improving tolerance to cat allergens [62]. Similarly, antihistamines such as bepotastine, loratadine, or levocetirizine have proven effective in alleviating nasal symptoms’ onset of action, efficacy, and safety of fexofenadine [68,89,90]. Furthermore, the combined antihistamine–decongestant fexofenadine–pseudoephedrine provided rapid symptom relief [72,73]. Additionally, reproxalap has shown both prophylactic and treatment efficacy in reducing ocular itching, tearing, and redness during an AEC challenge [77]. These findings underscore the versatility of AECs in assessing the efficacy of various anti-allergic therapies, extending beyond AIT.

Interestingly, one study validated distinct allergic rhinitis phenotypes (early-phase responders, protracted early-phase responders, dual responders, and low responders) across various allergens (birch, grass, ragweed, and house dust mite). Utilizing TNSS and PNIF as primary metrics, the study confirmed significant differences in allergic rhinitis responses during both early- and late-phase reactions, emphasizing the clinical relevance of these phenotypes in management [91].

AECs cannot be classified as medical devices (according to the Medical Device Directive 93/42/EEC) or medicinal products (according to Directive 2001/83/EC) [92]. AECs are utilized in Phase II clinical trials for AIT, where they have been validated as a robust tool for dose-finding, proof-of-concept studies, and the early demonstration of clinical effects [93,94]. However, despite their widespread use in Phase II trials, regulatory bodies like the European Medicines Agency (EMA) do not currently accept AEC data as a primary endpoint for pivotal phase III trials. This limitation underscores the need for further technical and clinical validation of AECs to meet the stringent regulatory requirements for product approval [14]. The EAACI has emphasized the importance of standardizing AEC protocols and has proposed the development of more rigorous validation methods to ensure that AEC data can be integrated into broader clinical and regulatory frameworks [8,93,95].

## 4. The Role of AECs in Asthma Research and Treatment Evaluation

In allergic asthma assessment, AECs play a crucial role in precisely inducing both early- and late-phase reactions, providing insights into how asthma symptoms develop and how AIT may mitigate them [96]. By offering controlled allergen exposure, AECs allow for the accurate measurement of lung function parameters like (FEV1 and PEFR, which are vital in assessing asthma responses) [26,97,98,99]. This controlled environment makes it easier to study the immediate and delayed effects of allergen exposure, which are difficult to capture in natural settings where factors like weather and pollution fluctuate. Additionally, AECs can potentially enable the real-time monitoring of asthma symptoms and inflammation markers (e.g., FeNO), allowing researchers to closely follow both early and prolonged asthmatic responses. In a study conducted using an AEC, mAb targeting Felis domesticus allergen 1 (Fel d 1) significantly prevented reductions in FEV1 and early asthmatic responses (EAR) in cat-allergic patients for up to 85 days following a single dose. This highlights the effectiveness of AECs in providing controlled conditions to evaluate the long-term therapeutic efficacy of treatments in managing allergic asthma symptoms over extended periods [97]. While AECs can simulate specific allergens like dust mites or pollen, they cannot perfectly mimic the full spectrum of environmental factors (e.g., pollutants) that may contribute to asthma exacerbations in the real world.

The studies conducted in AEC demonstrated the efficacy of air cleaners in reducing early and late asthmatic responses in cat-allergic patients, reinforcing the value of AECs in providing controlled conditions to assess bronchial responses to allergens, and further validated the use of AECs by showing that air cleaners significantly decreased airborne cat allergens, enabling safe and reproducible allergen exposure, which is crucial for asthma management and treatment evaluation [41,60]. The utility of AECs is further reinforced by a study involving HDM-allergic asthmatics, in which controlled allergen exposure within an AEC effectively triggered asthmatic symptoms. This demonstrates AEC’s efficacy in monitoring asthma treatments and assessing therapeutic responses [98].

## 5. Comparing AEC Testing with Nasal Allergen Challenges (NAC) and Field Assessments

Evidence suggests that the AEC method provides results that closely correlate with those obtained from NAC and real-world in-field symptom assessments [39,55,57,100,101,102]. Table 1 summarizes the key advantages and disadvantages of these methods, highlighting their utility and limitations in various clinical settings. AEC studies have consistently shown shorter durations for achieving reliable results than field studies, which require extended observation periods due to the unpredictability of natural allergen exposure. Additionally, AECs provide uniform exposure conditions, ensuring comparability between participants and studies.

Studies across different allergens, including timothy grass pollen, have demonstrated significant correlations between AEC results and in-field symptom measures such as TNSS or acoustic rhinometry [103]. Focusing on allergic conjunctivitis triggered via birch allergens, researchers compared two methods: the standard conjunctival provocation test and AEC. While both methods successfully triggered conjunctival responses, AECs produced results more akin to natural allergen exposure, showing greater reproducibility over repeated tests [57,104,105,106]. Another study evaluated birch-induced allergic rhinitis using three different methods: NACs, AECs, and exposure during the natural pollen season. The results indicated that the symptom responses elicited in AECs were similar to those observed during NAC and natural seasonal exposure, confirming that EECs can effectively replicate real-world allergen exposure [101]. Additionally, the effectiveness of AECs and NACs has been validated in other allergen contexts, such as in cat-allergic patients. Results obtained from both methods showed consistency in assessing the severity of allergic responses [107].

Overall, these studies demonstrate that AECs can provide a controlled yet accurate reflection of natural allergen exposure, offering an effective alternative to both NACs and field-based assessments [102,108].

## 6. Priming in AEC Studies: Importance and Implications

Priming refers to the phenomenon through which prior exposure to an allergen sensitizes an individual, leading to an enhanced immune response upon subsequent exposures. This effect is particularly significant in AEC studies, as it can influence the interpretation of drug efficacy.

It has been demonstrated that priming runs were necessary to elicit adequate symptomatic responses in participants exposed to Juniperus ashei pollen, emphasizing the role of priming in generating measurable effects for pharmacologic studies [19]. Also, in a different study, it was observed that pre-existing sensitivities to allergens like dust mites or grass pollen could accelerate and amplify symptom development upon controlled allergen exposure, indicating a “prepriming” phenomenon in individuals with seasonal allergic rhinitis [20]. Some studies have suggested that priming effects may not be evident in certain allergen exposure contexts. For example, in assessments of allergic conjunctivitis, baseline total ocular symptom scores measured 24 h after priming exposures were identified as late-phase reactions, rather than signs of enhanced sensitivity [109]. Similarly, a study on the conjunctival allergen challenge showed reproducible symptom scores across exposures without significant changes in sensitivity, further emphasizing the distinction between acute responses and late-phase reactions [57].

These studies underscore the need to account for priming effects in AEC designs, as unaddressed priming can lead to variability in baseline responses, overestimated drug efficacy, or confounded results.

## 7. Conclusions

AECs enhance the patient experience by offering a controlled and safe environment for allergen exposure. Unlike field studies, where allergen levels can vary unpredictably, AECs minimize the risk of severe allergic reactions by maintaining consistent allergen concentrations. Patients benefit from more predictable and manageable exposure, reducing the anxiety associated with uncontrolled allergen challenges. Furthermore, AECs are staffed by trained medical personnel, ensuring that immediate assistance is available in case of adverse reactions, thereby enhancing patient safety and confidence. Additionally, the ability to conduct studies within a single visit, rather than over several weeks or months, lowers the burden on participants and improves compliance. These advantages make AECs a more patient-friendly option for clinical trials, particularly for those who may struggle with long-term field studies.

AECs are valuable tools in researching airborne allergic diseases like rhinitis, conjunctivitis, and asthma, which have become widespread and impactful on society. These chambers provide high sensitivity and specificity, allowing for accurate symptom monitoring and analysis, which is particularly beneficial for evaluating the effectiveness of anti-allergic therapies [110]. While AECs are an invaluable tool in early-stage clinical research, their role in later-phase trials remains limited due to regulatory concerns [111]. Continued efforts to standardize and validate AEC protocols, alongside traditional field-based studies, will be critical in enhancing their utility for AIT and other therapeutics research [112]. The need to harmonize chamber techniques is critical, encompassing aspects such as defining batch-specific allergen profiles, standardizing the concentration of allergenic materials used, and implementing uniform symptom-scoring protocols. Additionally, the harmonization of facility parameters is essential to enable consistency and comparability across multicenter studies, ensuring reliable and reproducible results.

With further refinement, AECs hold the potential to play a more prominent role in the development and approval of new therapeutics and allergen immunotherapies.

## Figures and Tables

**Table 1:** Comparison of advantages and disadvantages of allergy assessment methods: allergen challenges (NAC/CPT) and field studies. Abbreviations: CPT = conjunctival provocation test; NAC = nasal allergen challenge.

Method	Advantages	Disadvantages
Allergen exposure chambers (AECs)	▪ Highly controlled allergen concentrations (compared to in-field studies) ▪ Reproducible and consistent exposure ▪ Simultaneous (potential) induction of rhinoconjunctivitis, asthma, and potential dermatitis flare-ups during the exposure, unlike unit-specific provocation tests (ocular, nasal, or bronchial) ▪ Allows precise measurement of both subjective and objective endpoints ▪ Reduction in external confounding variables such as weather and location differences (compared to field studies, but not with NAC and CPT) ▪ Can be suitable for clinical settings where control and consistency are crucial (after standardization and validation), such as evaluating early-stage clinical trials and allergy mechanisms	▪ A limited number of AECs worldwide ▪ High cost due to specialized equipment and trained personnel ▪ Not currently accepted as a primary endpoint for pivotal phase III studies by regulatory bodies ▪ Need for further standardization and validation ▪ Need for harmonizing of chamber techniques
Nasal/ ocular allergen challenges	▪ Direct application of allergen in a controlled dose to nasal passages ▪ Highly controlled allergen concentrations, often with standardized extracts ▪ Allows the precise measurement of both subjective and objective endpoints ▪ Cost-effective compared to AECs ▪ Suitable for assessing individual sensitivity and specific responses ▪ Often accepted in clinical trials	▪ Focus solely on nasal or ocular responses ▪ Cannot simultaneously assess rhinoconjunctivitis, asthma, or potential dermatitis flare-ups
Field studies	▪ Provide the most natural exposure context, making them ideal for understanding real-world efficacy ▪ Real-world conditions make the results directly relevant to everyday patient experience ▪ Useful for understanding natural allergen exposures across seasons and locations ▪ Assessment of rhinoconjunctivitis, asthma, and potential dermatitis flare-ups	▪ High variability due to environmental factors (e.g., weather) ▪ Long study durations, as the assessment is continued for many days ▪ Lack of standardization reduces reproducibility ▪ Inconsistent exposure levels of allergens make it hard to compare studies ▪ Difficult to conduct longitudinal and consistent monitoring due to fluctuating conditions
